# High-Target Hemodiafiltration Convective Dose Achieved in Most Patients in a 6-Month Intermediary Analysis of the CONVINCE Randomized Controlled Trial

**DOI:** 10.1016/j.ekir.2023.08.004

**Published:** 2023-08-19

**Authors:** Robin W.M. Vernooij, C. Hockham, C. Barth, B. Canaud, K. Cromm, A. Davenport, J. Hegbrant, M. Rose, G.F.M. Strippoli, M. Török, M. Woodward, M.L. Bots, P.J. Blankestijn

**Affiliations:** 1 Department of Nephrology and Hypertension, University Medical Center Utrecht, Utrecht University, Utrecht, The Netherlands; 2 Julius Center for Health Sciences and Primary Care, University Medical Center Utrecht, Utrecht University, Utrecht, The Netherlands; 3 George Institute for Global Health, School of Public Health, Imperial College London, London, UK; 4 B. Braun Avitum AG, Medical Scientific Affairs, Melsungen, Germany; 5 Montpellier University, School of Medicine, Montpellier, France and Global Medical Office, FMC Deutschland, Bad Homburg, Germany; 6 Fresenius Medical Care Deutschland GmbH, Global Medical Office, Bad Homburg, Germany; 7 UCL Center for Nephrology, Royal Free Hospital, Division of Medicine, University College London, London, UK; 8 Division of Nephrology, Department of Clinical Sciences, Lund University, Lund, Sweden; 9 Charité Universitätsmedizin Berlin, corporate member of Freie Universität Berlin, Humboldt-Universität zu Berlin, Berlin, Germany; 10 Center of Internal Medicine and Dermatology, Department of Psychosomatic Medicine, Berlin Institute of Health, Berlin, Germany; 11 Department of Precision and Regenerative Medicine and Ionian Area, University of Bari, Bari, Italy; 12 School of Public Health, University of Sydney, Sydney, Australia; 13 Corporate Medical Office Diaverum, Malmö, Sweden; 14 The George Institute for Global Health, University of New South Wales, Sydney, Australia

**Keywords:** baseline characteristics, convection volume, hemodiafiltration, hemodialysis, kidney failure, randomized controlled trial

## Abstract

**Introduction:**

High convection volumes in hemodiafiltration (HDF) result in improved survival; however, it remains unclear whether it is achievable in all patients.

**Methods:**

CONVINCE, a randomized controlled trial, randomized patients with end-stage kidney disease 1:1 to high-dose HDF versus high-flux hemodialysis (HD) continuation. We evaluated the proportion of patients achieving high-dose HDF target: convection volume per visit of ≥23 l (range ±1 l) at baseline, month 3, and month 6. We compared baseline characteristics in the following 2 ways: (i) patients on target for all 3 visits versus patients who missed target on ≥1 visits and (ii) patients on target for all 3 visits or missing it once versus patients who missed target on ≥2 visits.

**Results:**

A total of 653 patients were randomized to HDF. Their mean age was 62.2 (SD 13.5) years, 36% were female, 81% had fistula vascular access, and 33% had diabetes. Across the 3 visits, 75 patients (11%), 27 patients (4%), and 11 patients (2%) missed the convection volume target once, twice, and thrice, respectively. Apart from diabetes, there were no apparent differences in patient characteristics between patients who always achieved the high-dose target (83%) and those who missed the target either once or more (17%) or twice or more (6%).

**Conclusion:**

Achieving high-dose HDF is feasible for nearly all patients in CONVINCE and could be maintained during the 6-month follow-up period. Apart from diabetes, there were no other indications for confounding by indication on multivariable analyses that may explain the potential survival advantage for patients receiving high-dose HDF.

## Graphical abstract

**Figure:**
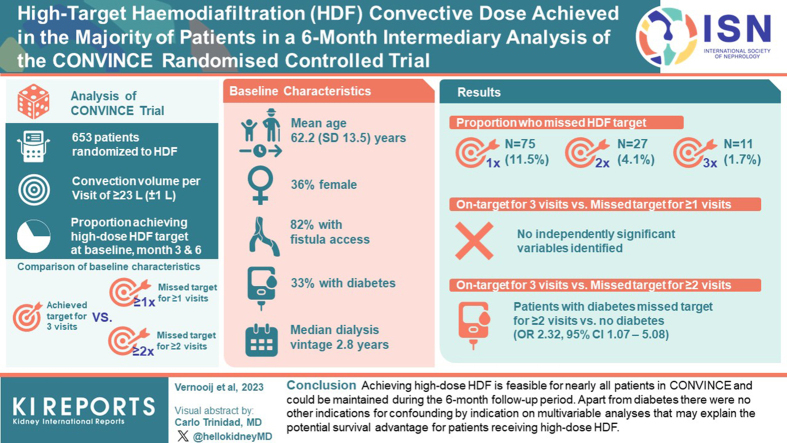

HDF has been found to reduce mortality in both observational studies^1^^,^^2^ and randomized controlled trials.^3^ This effect on mortality was most pronounced in the patients with end-stage kidney disease who received a higher convection volume (≥23 liters per 1.73 m^2^ body surface area per session in post-dilution HDF).^3^ However, the actual delivered convection volume in previous HDF trials revealed considerable variation, as a consequence of daily clinical practice and because patients were not randomized according to high-dose HDF treatment targets.^3^, ^4^, ^5^, ^6^ The possibility of confounding by indication, that is, only the healthier patients being able to achieve a higher convection volume and thus conferring a lower mortality risk, remains a point of discussion.

Some observational studies reported that patient characteristics associated with worse prognosis, including greater age, more comorbidities, and higher body mass index, affect the likelihood of achieving high HDF convection volumes.^7^, ^8^, ^9^ However, this has been contradicted by other observational studies where no differences were found in patient characteristics achieving high-dose HDF compared with those not achieving the target dose.^10^^,^^11^ In addition, in a recent randomized controlled trial, a high HDF convection volume (defined as >22 l per treatment) was achieved in 99% patients randomized to HDF, across different vascular access types, comorbidities, and baseline characteristics.^12^ However, this was in a younger and less comorbid dialysis population compared with the patients undergoing dialysis in Europe and North America. Owing to heterogeneity across studies in terms of patient, disease, and treatment characteristics, it remains unclear whether certain patient phenotypes consistently fail to achieve high-dose convection volumes.

The CONVINCE study was designed to determine the potential benefits and harms of high-dose HDF compared with high-flux HD with respect to mortality, cardiovascular diseases, hospitalizations, and patient-reported outcomes.^13^^,^^14^ A lower risk of all-cause mortality was found for the patients receiving high-dose, compared with high-flux HD, in the main analyses of CONVINCE.^14^ Hence, all evidence together suggests that high-dose HDF can result in a clinically important survival benefit^3^^,^^14^^,^^15^; however, it remains unclear whether every patient can receive high-dose HDF. In this article, we evaluate whether patients randomized to HDF were able to achieve a high-dose convection volume, and which, if any, baseline characteristics were associated with achieving this convection volume target.

## Methods

The protocol of the CONVINCE trial has been described elsewhere.^13^^,^^14^ In addition, CONVINCE is registered in the Netherlands National Trial Register (NTR 7138). In short, CONVINCE is an international, multicenter, prospective, open-label, randomized, controlled trial in adults (≥18 years) with end-stage kidney disease treated with high-flux HD for ≥3 months. Exclusion criteria were as follows: (i) severe nonadherence to the dialysis procedure and accompanying prescriptions, especially frequency and duration of dialysis treatment; (ii) life expectancy <3 months; (iii) HDF treatment <90 days before screening; (iv) anticipated living donor kidney transplantation <6 months after screening; and (v) evidence of any other diseases or medical conditions that may interfere with the planned treatment or compliance. Patients were recruited in 61 sites in the following 8 European countries: France, Germany, Hungary, Portugal, Romania, Spain, The Netherlands, and the United Kingdom.

### Study Intervention

Patients were allocated with a 1:1 ratio by a central block randomization scheme, stratified by center, to continuation of conventional high-flux HD or high-dose HDF. High-dose HDF was defined as a convection volume of ≥23 l (range ±1 l) with online production of substitution fluid, in post-dilution mode, and ultrapure bicarbonate-based dialysis fluid. In instances where the target convection volume was not initially achieved, a stepwise adjustment of dialysis prescription to achieve this target in 2 to 3 sessions was undertaken.^13^ The reference group received high-flux HD using ultrapure bicarbonate-based dialysis fluid as standard of dialysis care. In this article, we only include patients who received HDF. Patients were dialyzed with a variety of dialysis machines, from various manufacturers (Baxter Healthcare Corporation, Deerfield, IL; B Braun, Melsungen, Germany; Fresenius Medical Company, Bad Homberg, Germany; Nikkiso Company Limited, Tokyo, Japan, and Nipro Corporation, Osaka, Japan), and a variety of high-flux dialyzers from different manufacturers (Baxter Healthcare Corporation, Deerfield, IL; B Braun, Melsungen, Germany; Fresenius Medical Company, Bad Homberg, Germany, and Nipro Corporation, Osaka, Japan).

### Study Procedures

After randomization, patients continued thrice-weekly dialysis. Following baseline assessments, data were collected every 3 months until the end of the study. Data were collected during routine clinical practice, including demographics (e.g., year of birth, biological sex, ethnicity), relevant medical history, lifestyle information (e.g., smoking, alcohol use), concomitant medication, and current medical conditions. Cardiovascular history was defined as having angina pectoris, myocardial infarction, coronary stent or angioplasty procedure, coronary artery bypass graft, pacemaker, internal defibrillator, congestive heart failure, atrial fibrillation, cerebrovascular accident, transient ischemic attack, carotid endarterectomy, intermittent claudication, stent or angioplasty procedure of the arteries of the lower extremities, bypass surgery of the arteries of the lower extremities, abdominal aortic aneurysm, or stent or angioplasty procedure of the renal arteries.

Routine clinical measurements, including weight, systolic and diastolic blood pressures, and heart rate, were obtained before and after the dialysis session. Vascular access flow assessment was recorded at least twice a year. Laboratory measurements, including hemoglobin (mmol/l), single-pool Kt/V, creatinine (mg/dl), phosphorus (mmol/l), and C-reactive protein (mg/dl), were obtained. All assessments were performed by the local laboratory and were part of routine clinical practice. For patients randomized to HDF, information on the achieved convection volume and substitution volume was collected every 3 months. This concerned the achieved convection volume on the day of the study visit. Whenever this was missing, the most recent convection volume around this study visit was recorded.^13^

### Statistical Analyses

We tabulated the baseline characteristics for the patients who were randomized to HDF. We calculated the proportion of the patients who achieved a convection volume of ≥23 l (± 1l) at each of their first 3 scheduled visits (i.e., baseline, month 3, month 6). We compared baseline and treatment characteristics between patients who missed the target ≥1 visit and ≥2 visits with patients who achieved the target consistently or missed it only once, using Student *t* test, Pearson chi-square test, Fisher exact test, and Wilcoxon ranked sum test, depending on the nature and distribution of the variables. Four variables were log-transformed due to a skewed distribution, including time on dialysis (years), dialysis session duration (minutes), dialyzer urea clearance (Kt/V), and C-reactive protein (mg/l). Logistic regression models, combining age, sex, diabetes, cardiovascular history, height, post-dialysis weight, blood pressure, vascular access, and change in vascular access, were fitted to explore whether certain variables remained significant with missing the target after adjusting for others. These characteristics were selected based on previous studies and available data.^2^^,^^7^^,^^8^^,^^10^^,^^12^ A narrative synthesis on the clinical notes concerning the justification why the patients missed the high-dose convective volume target was constructed using extracted text information from the electronic patient study records. All statistical analyses were performed in R (version 3.5.1), and a two-sided *P* value of <0.05 conferred statistical significance.

## Results

### Patient Population

In total, 653 patients were randomized to HDF and completed at least 3 visits (Table 1). The mean age of the patients was 62.2 (SD 13.5) years, and 36.1% of the patients were female. One-third of the patients had diabetes mellitus, 43.1% had a history of cardiovascular disease at baseline, and the mean body mass index was 27.3 kg/m^2^ (SD 5.5). The median dialysis vintage was 2.8 (interquartile range: 1.8–6.5) years. Most patients (81%, *n* = 532) had a fistula as vascular access at baseline. During follow-up, 10% of the patients changed the type of vascular access.

**Table 1:** Baseline characteristics of the included patients

Characteristics	HDF (*N* = 653)
Age (yr), mean (SD)	62.23 (13.50)
Female (%)	236 (36.1)
History of CHD (%)	124 (19.0)
History of CVD (%)	281 (43.1)
Diabetes mellitus (%)	216 (33.1)
Body mass index (kg/m^2^), mean (SD)	27.3 (5.5)
Height (cm), mean (SD)	167.84 (9.64)
Weight (kg), mean (SD)	76.99 (16.90)
Body surface area (m^2^), mean (SD)	1.86 (0.22)
Current smoking (%)	94 (14.5)
Current alcohol consumption	163 (25.2)
Dialysis vintage (yr), median (IQR)	2.83 [1.37, 6.46]
Previous renal transplant (%)	90 (13.8)
Systolic BP (mm Hg) predialysis, mean (SD)	141.41 (21.81)
Diastolic BP (mm Hg) predialysis, mean (SD)	73.31 (13.72)
Heart rate (beat/min) predialysis, mean (SD)	72.52 (11.10)
Systolic BP (mm Hg) postdialysis, mean (SD)	137.01 (22.30)
Diastolic BP (mm Hg) postdialysis, mean (SD)	71.55 (14.04)
Heart rate (beat/min) postdialysis, mean (SD)	71.82 (12.85)
Vascular access (%)	
Fistula	532 (81.5)
Catheter	88 (13.5)
Graft	33 (5.1)
At least one change in vascular access (%)	65 (10.0)
Number times vascular access changed (%)	
0	588 (90.1)
1	53 (8.1)
2	12 (1.8)
Duration of dialysis session (min), median (IQR)	240.0 [240.0, 246.0]
Net ultrafiltration rate (ml), mean (SD)	2199.4 (1144.8)
Extracorporeal blood flow rate (ml/min), mean (SD)	369.95 (54.52)
Dialysis single-pool Kt/V, median (IQR)	1.61 [1.45, 1.85]
Hemoglobin (mmol/l), mean (SD)	7.02 (0.76)
Phosphorus (mmol/l), mean (SD)	1.58 (0.49)
C-reactive protein (mg/dl), median (IQR)	0.49 [0.22, 1.08]
Creatinine (mg/dl) predialysis, mean (SD)	8.37 (2.38)

BP, blood pressure; CHD, coronary heart disease; CVD, cardiovascular disease; HDF, hemodiafiltration; IQR, interquartile range; yr, year.

### Achieved Convection Volume at Patient Level

**Figure 1:**
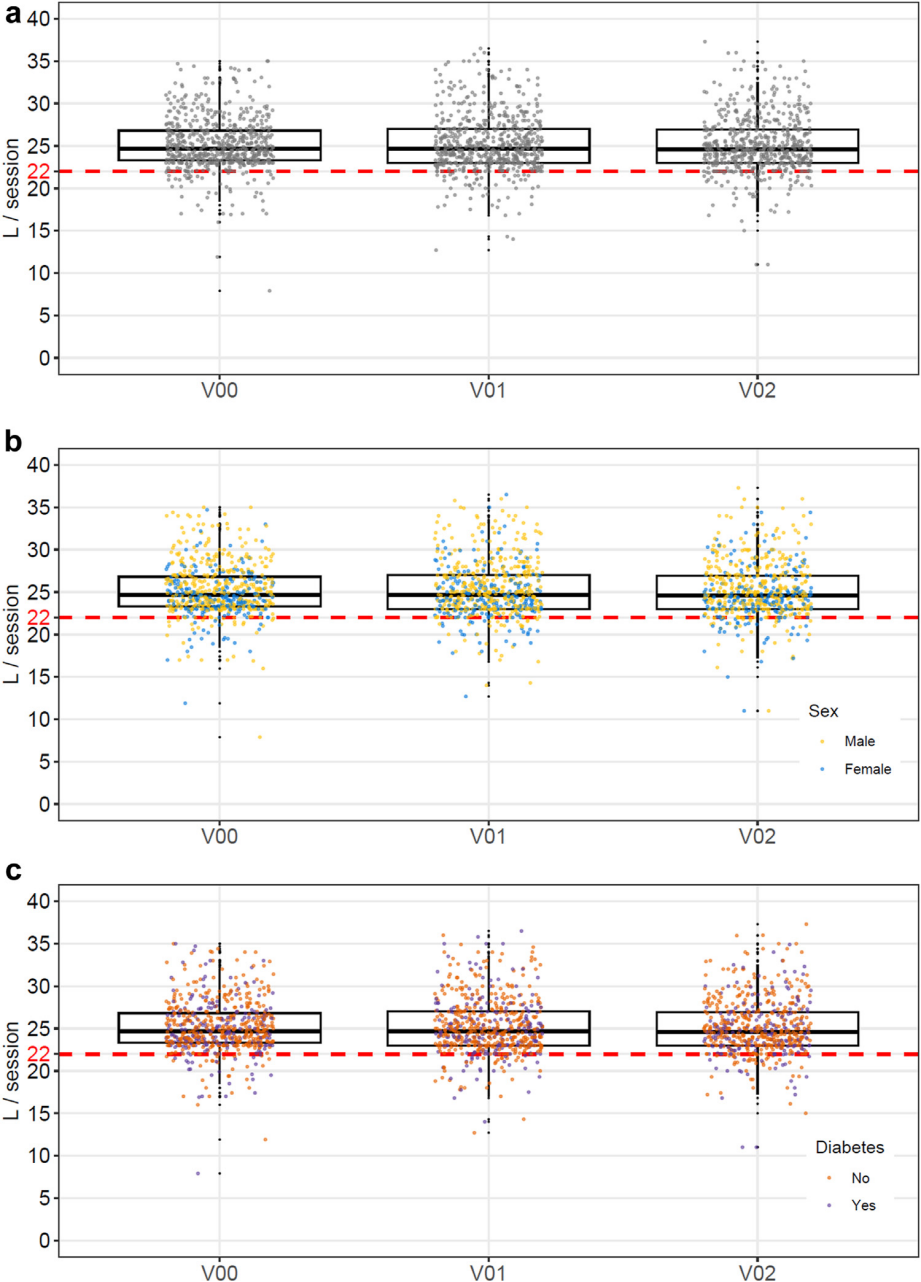
(a) Achieved convective volume for all patients during follow-up. (b) Achieved convective volume stratified for males and females. (c) Achieved convective volume stratified for patients with and without diabetes.

The high-dose HDF target was missed once by 75 patients (11.5%), twice by 27 (4.1%), and thrice by 11 (1.7%) (Table 2, Figure 1a–c). For the patients missing the target once, no differences were observed in terms on which visit (i.e., baseline, visit 1, or visit 2) this occurred. Across 61 dialysis centers in 7 countries, using a variety of dialysis machines and dialyzers, there were no significant differences in achieving target convective exchanges.

**Table 2:** Number of patients in whom the target convection volume was not achieved on one or more visits

No. of visits	*N* (%) patients missing target
One or more	113 (17.3)
One	75 (11.5)
Visit 0	24 (32)
Visit 1	23 (30.7)
Visit 2	28 (37.3)
Two	27 (4.1)
Three	11 (1.7)

### On High-Dose Target for All Three Visits Versus Missing the Target on ≥1 Visits

There were few differences in baseline characteristics between the patients who achieved their convective volume target for all 3 visits and those who missed the target on ≥1 visits (Table 3). Postdialysis systolic blood pressure was lower (132 [22] mm Hg vs. 138 [22] mm Hg) (*P* = 0.004), and predialysis heart rate was higher (75 [12] beats/min vs. 72 [11] beats/min) (*P* = 0.036) for patients who missed the target on ≥1 visits. Extracorporeal blood flow rate was lower (354 [57] ml/min vs. 373 [54] ml/min) (*P* < 0.001) for patients who missed the target on ≥1 visits. Patients who changed vascular access missed the target on ≥1 visits more often than patients who did not change (*P* = 0.020). In the multivariable regression, no independently significant variables were identified (Table 4).

**Table 3:** Baseline characteristics comparing patients who achieved the high-dose target versus patients who missed the high-dose target

Characteristics	On target for all visits	Below target for ≥1 visits	*P*-value	On target for all visits or missing it once	Below target for ≥2 visits	*P*-value
*N*	540	113		615	38	
Age (yr)	62.40 (13.52)	61.41 (13.41)	0.478	62.18 (13.70)	63.00 (9.68)	0.716
Women (%)	187 (34.6)	49 (43.4)	0.079	216 (35.1)	20 (52.6)	0.029
History of CHD %)	107 (19.8)	17 (15.2)	0.255	117 (19.0)	7 (18.9)	0.987
History of CVD (%)	235 (43.5)	46 (41.1)	0.634	267 (43.4)	14 (37.8)	0.506
Diabetes mellitus (%)	171 (31.7)	45 (40.2)	0.082	197 (32.0)	19 (51.4)	0.015
Height (cm)	168.23 (9.39)	166.0 (10.17)	0.025	167.99 (9.53)	165.34 (11.19)	0.100
Weight (kg)	77.09 (16.70)	76.52 (17.91)	0.745	76.91 (16.77)	78.37 (19.03)	0.605
Body surface area (m^2^)	1.86 (0.22)	1.84 (0.22)	0.262	1.86 (0.22)	1.85 (0.22)	0.815
Smoking (%)			0.702			0.188
Never	279 (51.8)	62 (55.9)		319 (52.0)	22 (61.1)	
Current	80 (14.8)	14 (12.6)		87 (14.2)	7 (19.4)	
Past	180 (33.4)	35 (31.5)		208 (33.9)	7 (19.4)	
Alcohol consumption (%)			0.249			0.397
Never	291 (54.1)	51 (46.8)		324 (53.0)	18 (50.0)	
Current	135 (25.1)	28 (25.7)		156 (25.5)	7 (19.4)	
Past	112 (20.8)	30 (27.5)		131 (21.4)	11 (30.6)	
Dialysis vintage (yr)	2.96 [1.40, 6.49]	2.60 [0.89, 5.41]	0.152	2.83 [1.37, 6.35]	2.91 [0.92, 8.45]	0.746
Previous renal transplant (%)	69 (12.8)	21 (18.6)	0.103	83 (13.5)	7 (18.4)	0.393
Systolic BP (mm Hg) predialysis	141.46 (22.01)	141.16 (20.92)	0.893	141.09 (21.68)	146.55 (23.56)	0.134
Diastolic BP (mm Hg) predialysis	73.26 (13.63)	73.57 (14.19)	0.829	73.12 (13.64)	76.47 (14.78)	0.143
Heart rate (beat/min) predialysis	72.10 (10.89)	74.54 (11.93)	0.036	72.36 (11.13)	75.06 (10.34)	0.158
Systolic BP (mm Hg) postdialysis	138.14 (22.19)	131.49 (22.14)	0.004	137.21 (22.31)	133.64 (22.16)	0.351
Diastolic BP (mm Hg) postdialysis	71.80 (14.26)	70.33 (12.90)	0.317	71.54 (14.05)	71.69 (14.04)	0.949
Heart rate (beat/min) postdialysis	71.55 (12.65)	73.19 (13.80)	0.230	71.71 (12.86)	73.74 (12.81)	0.373
Vascular access (%)			0.128			0.99
Fistula	444 (82.2)	88 (77.9)		501 (81.5)	31 (81.6)	
Catheter	73 (13.5)	15 (13.3)		83 (13.5)	5 (13.2)	
Graft	23 (4.3)	10 (8.9)		31 (5.0)	2 (5.3)	
At least one change in vascular access (%)	47.0 (8.7)	18.0 (15.9)	0.020	60 (9.8)	5 (13.2)	0.572
Number times vascular access changed (%)			0.037			0.518
0	493 (91.3)	95 (84.1)		555 (90.2)	33 (86.8)	
1	37 (6.9)	16 (14.2)		49 (8.0)	4 (10.5)	
2	10 (1.9)	2 (1.8)		11 (1.8)	1 (3%)	
Duration of dialysis session (min)	240.0 [240.0–246.5]	240.0 [240.0–245.0]	0.721	240.0 [240.0–246.0]	240.0 [240.0–245.0]	0.571
Net ultrafiltration rate (ml)	2178.5 (1157.4)	2298.4 (1082.4)	0.31	2191.3 (1148.5)	2331.5 (1089.5)	0.464
Extracorporeal blood flow rate (ml/min)	373.18 (53.48)	354.41 (56.97)	<0.001	372.04 (54.43)	335.27 (43.66)	<0.001
Dialysis single-pool Kt/V	1.61 [1.46–1.85]	1.60 [1.42–1.85]	0.988	1.61 [1.45, 1.85]	1.64 [1.53, 1.91]	0.212
Hemoglobin (mmol/l)	7.00 (0.73)	7.12 (0.91)	0.119	7.03 (0.76)	6.89 (0.81)	0.296
Phosphorus (mmol/l)	1.56 (0.47)	1.68 (0.57)	0.030	1.57 (0.48)	1.79 (0.3)	0.011
C-reactive protein (mg/l)	0.46 [0.20–1.10]	0.55 [0.31–0.94]	0.210	0.49 [0.21, 1.08]	0.42 [0.25, 0.75]	0.998
Creatinine (mg/dl) predialysis	8.37 (2.34)	8.37 (2.61)	0.99	8.36 (2.39)	8.52 (2.26)	0.716

BP, blood pressure; CHD, coronary heart disease; CVD, cardiovascular diseases; IQR, interquartile range.

Mean (SD) and median [IQR] for continuous variables; *N* (%) for categorical variables.

**Table 4:** Multivariable model comparing patients who missed the high-dose target versus patients who achieved the target

Characteristics	Below target for ≥1 visits vs. never missing the target OR (95% CI)	Below target for ≥2 visits vs. never missing the target once or less OR (95% CI)
Age (per yr)	0.99 (0.98–1.01)	1.02 (0.99–1.05)
Sex		
Men	Ref.	Ref.
Women	1.11 (0.64–1.93)	2.08 (0.84–5.25)
Diabetes		
No	Ref.	Ref.
Yes	1.61 (0.99–2.60)	2.32 (1.07–5.08)
Cardiovascular history		
No	Ref.	Ref.
Yes	0.90 (0.57–1.41)	0.69 (0.32–1.43)
Height (per cm)	0.98 (0.95–1.01)	0.98 (0.93–1.03)
Postdialysis weight (per kg)	1.00 (0.99–1.01)	1.01 (0.99–1.03)
Systolic blood pressure (per mm Hg)	1.00 (0.98–1.01)	1.01 (0.99–1.03)
Diastolic blood pressure (per mm Hg)	1.00 (0.98–1.03)	1.03 (0.99–1.06)
Vascular access		
Fistula	Ref.	Ref.
Catheter	0.88 (0.46–1.61)	0.70 (0.22–1.79)
Graft	1.72 (0.69–4.00)	0.77 (0.11–3.17)
Change in vascular access		
No	Ref.	Ref.
Yes	1.83 (0.93–3.46)	1.69 (0.52–4.58)

OR, odds ratio; Ref., reference.

### On High-Dose Target for All 3 Visits or Missing It Once Versus Missing the Target on ≥2 Visits

Results comparing patients who achieved the high-dose target for all 3 visits or missed it only once with patients who missed the target on ≥2 visits are found in Table 3. Women (53% vs. 35%, *P* = 0.029) and patients with diabetes (51% vs. 32%, *P* = 0.015) were more often below target on ≥2 visits. No differences in vascular access and dialysis session duration were observed, but extracorporeal blood flow rate was lower (335 [44] ml/min vs. 372 [54] ml/min) (*P* < 0.001) for patients who missed the target on ≥2 visits. In the multivariable analyses, patients with diabetes missed the target ≥2 visits more often than patients without diabetes (odds ratio: 2.32, 95% CI: 1.07–5.08).

### Narrative Synthesis

In the clinical notes, reasons why patients missed the convective volume target were only reported for 16 patients, where for the other patients missing target remained missing. When reasons were given for missing the target, problems with the dialyzer or blood flow (*n* = 11), vascular access (*n* = 3), and shorter dialysis session (*n* = 2) were reported.

## Discussion

### Summary of Main Findings

Achieving a high-dose convection volume in HDF treatment is feasible for most patients and, most importantly, could be maintained during the present trial period. We did not identify a certain phenotype of patients who consistently missed the convective volume target, although patients with diabetes and women seem to have a higher risk of missing the target. Across all centers and countries, no differences in achieving target convective exchanges were identified. Furthermore, patients with a vascular access that provides lower extracorporeal blood flow rates miss the high-dose target more often. Achieving high-dose HDF can be achieved using a variety of dialysis machines and high-flux dialyzers and is not dialysis center practice dependent. Hence, we will be able to investigate in the CONVINCE study whether high-dose HDF is superior to high-flux HD with respect to mortality, cardiovascular diseases, hospitalizations, and patient-reported outcomes.

### Comparison With Previous Research

A limited number of studies have investigated whether, and more importantly in which patients, a high-dose HDF target can be achieved. Some observational studies state that patient characteristics associated with worse prognosis, including age, comorbidities, and body mass index, affect the likelihood of achieving high HDF convection volumes.^7^, ^8^, ^9^ Our results do not support that patient factors have a substantial effect on failing to achieve high volume HDF targets. A high convection volume (defined as >22 l per treatment) was achieved in 99% patients randomized to HDF in a recent randomized controlled trial, across different vascular access types, comorbidities, and baseline biochemical variables.^12^ These contradictions might also be explained by differences across study populations. For example, the studies by Neri *et al.*^7^ and Guedes *et al.*^12^ included a relatively young patient population (mean age of 55.8 [13.8] years and 52.6 [15.9] years, respectively), with lower rates of cardiovascular history (coronary arterial disease: 7.7% and 14.4%, respectively).

In a cross-sectional analysis of the CONTRAST trial, few patient characteristics were associated with achieving adequate convection volume, apart from effective extracorporeal blood flow rate and treatment time. Hence, a personalized approach on achieving the high-dose HDF is suggested.^16^ Since the CONTRAST trial, more education on how to achieve a high convection dose was developed, facilitating the implementation of high-dose HDF. Furthermore, in a large (*n* = 3315) cross-sectional analysis in 6 European countries, extracorporeal blood flow rate, treatment time, and filter surface area are factors suggested to play an important role in achieving high-volume HDF.^17^ A higher extracorporeal blood flow rate, but not treatment time, was also a significant factor related to achieving the high-dose HDF target in our study.

### Implications for Clinical Practice

Confounding by indication does not seem to be the explanation for the potential survival benefit in HDF. Most patients can achieve a high-dose HDF target. The CONVINCE trial protocol proposes the steps required to obtain the high-dose target.^13^^,^^14^ CONVINCE is a large pragmatic trial, with no strict inclusion and exclusion criteria, and has been designed to determine the benefits and harms of high-dose HDF versus high-flux HD. In addition to efficacy and safety, patient perspectives along with cost-effectiveness will be analyzed. A lower risk of all-cause mortality was found for the patients receiving high-dose, compared with high-flux HD, in the main analyses of CONVINCE.^14^ Achieving high-dose HDF is feasible for nearly all patients in CONVINCE and could be maintained during follow-up.

### Strengths and Limitations

Our study has strengths and limitations. We have included a large number of patients across different countries and centers in Europe. The CONVINCE study and the way of obtaining a high-dose HDF target are strictly protocolled.^13^^,^^14^ We have investigated this issue in 3 different visits with a maximum follow-up of 6 months. Hence, we cannot draw conclusions regarding achieving convection volumes in the longer term. The reasons for missing the high-dose target were often not well recorded in the clinical notes, and so could not be analyzed accordingly. Finally, given the pragmatic design of CONVINCE, limited information on the HDF machines and dialyzers was collected. Hence, we could not explore whether there were any differences across the different dialyzers and machines. However, across the different countries and centers, using a variety of dialysis machines and dialyzers, there were no significant differences in achieving target convective exchanges.

## Disclosure

CB is an employee of B. Braun Avitum AG. BC was a former employee and acting as scientific consultant for Fresenius Medical Care Deutschland GmbH. KC is an employee of Fresenius Medical Care Deutschland GmbH. AD receives fees from Fresenius Medical Company and Nipro Corporation for speaking at scientific meetings and attending advisory groups. JH serves on the Board of Directors of LundaTec AB and NorrDia AB and provides consultancy services to Triomed AB. MT is an employee of Diaverum. MW has been a recent consultant to Amgen and Freeline. PJB received funding from the European Commission (Horizon 2020 grant no 754803-2), speaker fee from Fresenius, and consultancy fee from Medtronic; in all cases, the funds were transferred to the institution. RV, CH, MR, GS, and MLB have no conflict of interest to report.

## Supplementary Material

Supplementary File (PDF)

**STROBE Statement.**
